# Length of Barrett’s segment predicts failure of eradication in radiofrequency ablation for Barrett’s esophagus: a retrospective cohort study

**DOI:** 10.1186/s12876-018-0799-6

**Published:** 2018-05-21

**Authors:** Tyler Luckett, Chaitanya Allamneni, Kevin Cowley, John Eick, Allison Gullick, Shajan Peter

**Affiliations:** 1 Department of Gastroenterology and Hepatology, University of Alabama at Birmingham, 1720 2nd Avenue South, BDB 380, Birmingham, AL 35294 USA; 2 University of North Carolina Internal Medicine Residency, University of North Carolina, Carolina, North, USA

**Keywords:** Barrett’s esophagus, Radiofrequency ablation, Endoscopy, Esophagus

## Abstract

**Background:**

We aim to investigate factors that may contribute to failure of eradication of dysplastic Barrett’s Esophagus among patients undergoing radiofrequency ablation treatment.

**Methods:**

A retrospective review of patients undergoing radiofrequency ablation for treatment of Barrett’s Esophagus was performed. Data analyzed included patient demographics, medical history, length of Barrett’s Esophagus, number of radiofrequency ablation sessions, and histopathology. Subsets of patients achieving complete eradication were compared with those not achieving complete eradication.

**Results:**

A total of 107 patients underwent radiofrequency ablation for Barrett’s Esophagus, the majority white, overweight, and male. Before treatment, 63 patients had low-grade dysplasia, and 44 patients had high-grade dysplasia or carcinoma. Complete eradication was achieved in a majority of patients (57% for metaplasia, and 76.6% for dysplasia). Failure of eradication occurred in 15.7% of patients. The median number of radiofrequency ablation treatments in patients achieving complete eradication was 3 sessions, compared to 4 sessions for failure of eradication (*p* = 0.06). Barrett’s esophagus length of more than 5 cm was predictive of failure of eradication (*p* < 0.001).

**Conclusions:**

Radiofrequency ablation for dysplastic Barrett’s Esophagus is a proven and effective treatment modality, associated with a high rate of complete eradication. Our rates of eradication from a center starting an ablation program are comparable to previously published studies. Length of Barrett’s segment > 5 cm was found to be predictive of failure of eradication in patients undergoing radiofrequency ablation.

## Background

Barrett’s Esophagus (BE) is a condition in which the stratified squamous epithelium that lines the distal esophagus is replaced by metaplastic columnar epithelium that predisposes to the development of dysplasia and adenocarcinoma [1]. Esophageal adenocarcinoma (EAC) incidence has been on the rise, most drastically in the Caucasian segment of the American population [2, 3]. Therefore, it is important to adequately address dysplastic precursor lesions to EAC. A relatively recent addition to gastrointestinal endoscopy is radiofrequency ablation (RFA) using the HALO system (BARRX Medical, Inc., Sunnyvale, CA, USA), which has been shown to be safe and effective for the treatment of BE, including low-grade dysplasia (LGD) and high-grade dysplasia (HGD) [4–6]. Not only is RFA associated with decreased neoplastic progression compared to surveillance endoscopy [7, 8], a recent meta-analysis of the literature showed a pooled complete eradication of intestinal metaplasia (CE-IM) rate of 78% (95% CI 70–86%) and complete eradication of dysplasia (CE-D) rate of 91% (95% CI 87–95%) [9].

Despite high rates of eradication, as many as one-third of patients experience recurrence after complete eradication [10]. Some cited predictors of recurrence are older age, non-Caucasian race and longer length of pretreatment BE [11, 12]. Additionally, some patients do not respond to RFA or require multiple sessions to obtain complete eradication. While some have not been able to determine any significant predictors of response to therapy [13], others have found that active reflux disease, longer history of dysplasia, increased hiatal hernia size as well as increased length of BE are all predictors of RFA failure [14–16].

The success of RFA is such that it has become integrated at many large institutions in combination with resection techniques, such as endoscopic mucosal resection (EMR) and endoscopic submucosal dissection (ESD), which are needed to remove macroscopically visible lesions [17]. Given this increased use, it is vital to determine which patients may be at high risk for not responding to RFA and thus neoplastic progression. The current literature is conflicting, as studies that have found predictors of RFA failure differ in their results. For instance, Lyday et al. found CE-IM to be inversely related to BE length [15], while van Vilsteren et al. did not find BE length to be statistically significant [14], leading to the conclusion that further investigation is warranted.

The goals of this study were as follows: (1) to determine factors that may predict failure of CE-IM and CE-D in patients treated with RFA, and (2) to report the rates of CE-IM and CE-D at a large institution that recently began offering RFA and compare them to those previously published in the literature.

## Methods

### Study design

After the study was reviewed and approved by the University of Alabama at Birmingham Institutional Review Board (IRB), a retrospective review of consecutive patients undergoing RFA between December 2009–February 2015, for treatment of Barrett’s Esophagus at the University of Alabama at Birmingham (UAB) was performed. All study participants provided informed written consent prior to study enrollment. Data was entered and stored in a de-identified spreadsheet. Data abstracted for analysis included patient demographic characteristics, medical history, pathological findings, endoscopic findings, endoscopic procedures, adverse events, treatment, and biopsies with histopathology findings on surveillance. A standard four quadrant biopsy protocol based on the Seattle protocol was used for sampling [18]. As part of this protocol, targeted biopsy using narrow band imaging was performed. All biopsies were examined by the same experienced GI pathologist, and were reviewed again by a separate pathologist for documentation of consensus. Histopathology was graded and classified as high grade dysplasia, low grade dysplasia, or intestinal metaplasia. Both endoscopic inspection and biopsy results were used to determine which patients needed additional rounds of RFA to try to achieve CE. The biopsy protocol at our institution was as follows: We would continue RFA sessions until BE appeared endoscopically cleared, and then biopsies would be obtained at that time. Patients in our study required between two to ten RFA sessions to achieve complete eradication (mean = 3 sessions), and after any number of RFA sessions, if the patient appeared to have endoscopic clearance of BE, then biopsies would be obtained at that time to document complete eradication. Similarly, if a patient had an endoscopically visible lesion that needed targeted biopsy, then biopsies would be obtained at that time. Based on the results of mucosal biopsies after endoscopic treatment, patients were then divided based on histopathology into complete eradication (CE) of dysplasia (CE – D) or intestinal metaplasia (CE – IM). Subsets of patients achieving CE were compared with those not achieving CE. Those patients with mucosal biopsies demonstrating persistent dysplasia or intestinal metaplasia after treatment with RFA were considered failure of CE. Thus, failure was based upon histology, not endoscopy. Patients were considered lost to follow-up if post-treatment biopsies were not obtained.

### Procedure description

Patients were placed in the decubitus supine position. All procedures were performed with patients under monitored anesthesia care (MAC). Measurements of BE were done using the Prague Classification [19]. Patients underwent ablation using the circumferential device (HALO^360^ system) or a focal device (HALO^90^ both from Covidien GI Solutions) according to the extent of disease and investigator preference, as previously described. Subsequent ablation sessions were performed every 2 months, until complete endoscopic and histological eradication of Barrett’s Esophagus. At each ablation session, if any visible nodular lesions were identified, these were first treated with Endoscopic Mucosal Resection (EMR) using the Band ligation with the Duette Multi-Band Mucosectomy Device (Wilson-Cook, Winston-Salem, NC, USA) as previously described [20]. Then, the gastro-esophageal junction was ablated circumferentially, irrespective of its endoscopic appearance. Our protocol for ablation therapy has been previously described [21]. In more detail, the protocol for circumferential ablation and focal ablation included endoscopy with visual inspection, reading landmarks, sizing balloon, selection of ablation type, first pass ablation, clearing the face, and then second pass ablation. Focal ablation RFA was performed for treating shorter segments or islands of tongues of BE. Energy was delivered at settings of 12 J/cm2. A similar second pulse of energy was given after cleaning the electrode. Each target area received a total of 4 energy ablations for focal ablation and 2 for circumferential ablation respectively. The average length of each RFA treatment was 15.6 min.

CE-IM and CE-D were defined as complete eradication of IM and dysplasia, respectively, as documented by histopathology from mucosal biopsy obtained by white-light endoscopy (GIF-HQ190 Olympus, Tokyo, Japan). Time to CE-IM or CE-D was measured from the date of first RFA to the first follow-up EGD with normal histopathology reported for biopsy specimens. Recurrence was defined as the presence of IM or dysplasia in standard surveillance biopsies. The neosquamocolumnar junction was assessed in every case by white-light endoscopy with biopsies. For surveillance, 4-quadrant biopsies were performed at 1 cm intervals of the original extent of the Barrett’s Esophagus, starting at 1 cm proximal to the top of the gastric folds. In addition, any suspicious visible lesions were targeted, biopsied, and placed in separate jars. Remission of intestinal metaplasia/ dysplasia was confirmed with endoscopic findings and the four quadrant biopsy protocol.

### Statistical analysis

Unadjusted univariate and bivariate comparisons were made, utilizing chi-square or Fisher exact test for categorical variables and two tailed t-tests or Wilcoxon Rank Sums for continuous variables, where appropriate. Negative binomial logistic regression was used to model predictors of failure for CE-IM and CE-D utilizing stepwise selection. Significance was determined by a *p* < 0.05. All statistical analysis was performed using SAS 9.4 (SAS Institute Inc., Cary, NC).

## Results

A total of 107 patients underwent RFA for BE. Overall, 96.3% (*n* = 103) of the patients were white, and 86.9% (*n* = 93) were male. The median age was 64 years (range 58–72 years), and the mean length of Barrett’s esophagus was 6.7 cm (range 2-8 cm, median 5 cm). Most patients were overweight, with mean BMI 29.1 (range 25.5–32.6). On average, each patient underwent 3 (range 2–10, median 3) RFA procedures. The median time until CE-IM was 238 days (119–474) and the median time until CE-D was 251 days (133–525). There were 20 patients (15.7%) who did not obtain post-treatment biopsies, and were considered lost to follow up. Of the patients included in the study, 41.1% had HGD, and 58.9% had LGD. After RFA treatment, 57.0% of patients achieved CE-IM, and 76.6% achieved CE-D. 4.7% of patients progressed to esophageal adenocarcinoma. The average time to progression from dysplasia to adenocarcinoma was 170 days. The initial pathology for all patients that progressed to esophageal adenocarcinoma was HGD. Focal ablation was performed only for shorter segments of BE, as this tends to be more effective than circumferential ablation for these lesions. Comparing eradication rates between focal and circumferential ablation was not the main study objective, so our study did not directly compare differences in circumferential ablation versus focal ablation. Also, many longer segment BE lesions initially treated with circumferential ablation were later followed up with focal ablation, making it difficult to directly compare circumferential and focal ablation. There were no statistically significant differences in rates of CE-IM or CE-D for patients with HGD versus those with LGD. There were no statistically significant differences in BE segment length in patients with HGD (mean 6.2 ± 4.2 cm) versus LGD (mean 5.3 ± 3.8 cm).

Independent predictors of failure to achieve CE-IM [see Table 1] included age > 64 years, (OR: 2.6, (1.20–5.79); *p* < 0.02), and having a BE segment length greater than 5 cm (OR: 4.03(1.78–9.09); *p* < 0.001). On adjustment, both age (OR: 4.508, (1.72–11.84), *p* < 0.0022) and length of segment (OR: 7.064, (2.62–19.06), p < 0.001) remained significant predictors of failure to achieve CE-IM.

**Table 1:** Factors Affecting Complete Eradication of Intestinal Metaplasia

	All Patients^a^		Complete Eradication of Intestinal Metaplasia		Incomplete Eradication of Intestinal Metaplasia		
	107	100.0%	61	57.0%	46	43.0%	*p*-value^b^
Patient Characteristic							
Race							
White	103	96.3%	59	96.7%	44	95.7%	0.773
Other	4	3.7%	2	3.3%	2	4.3%	
Sex							
Male	93	86.9%	51	83.6%	40	86.9%	0.242
Female	14	13.1%	10	16.4%	6	13.1%	
Age	^c^64	(58-72)	^c^ 63	(57–72)	^c^ 67	(59–76)	0.117
BMI	^c^ 29.1	(25.5–32.6)	^c^ 30.9	(26.5–33.1)	^c^ 28.3	(24.5–30)	0.077
Dysplasia							
HGD	44	41.1%	24	39.3%	20	43.5%	0.667
LGD	63	58.9%	37	60.7%	26	56.5%	
Comorbidities							
GERD	75	70.1%	30	49.2%	32	69.6%	0.339
Hyperlipidemia	26	24.3%	17	27.9%	11	23.9%	0.322
Diabetes	25	23.4%	15	24.6%	10	21.7%	0.730
Hypertension	59	55.1%	30	49.2%	31	67.4%	0.154
History of Tobacco Usage							
Yes	60	56.1%	30	49.2%	29	63.0%	0.225
No	33	30.8%	21	34.4%	13	28.3%	
Unknown	14	13.1%	10	16.4%	4	8.7%	
Endoscopic Treatments Received							
EMR	24	22.4%	16	26.2%	8	17.4%	0.326
Length							
Median, IQR	5	(2–7)	3	(2–7)	7	(2–8)	< 0.001
</= 5 cm	64	59.8%	44	72.1%	20	43.4%	< 0.001
> 5 cm	43	40.2%	17	27.9%	26	56.6%	
Number of RFA’s							
Median, IQR	3	(2–5)	3	(2–5)	4	(2–5)	0.023
≤ 3	62	57.9%	41	67.2%	21	45.7%	0.008
> 3	45	42.1%	20	32.8%	25	54.3%	

^a^Data presented as N (%) or median (IQR)

^b^*p*-value ≤0.05 is significant

^c^average; not total number of patients

Independent predictors of failure to achieve CE-D [see Table 2] included having hypertension (OR: 3.33(1.21–9.17); *p* = 0.02), and having a BE segmental length greater than 5 cm (OR: 2.60 (1.04–6.51); *p* = 0.04). On adjustment, both hypertension (OR 3.86; 1.32–11.31, *p* < 0.01) and length of segment (OR 3.08; 1.12–8.46, *p* < 0.03) remained significant predictors of failure to achieve CE-D (see Table 3). The number of patients who developed adenocarcinoma was very small, so no independent predictors were identified.

**Table 2:** Factors Affecting Complete Eradication of Dysplasia

	All Patients^a^		Complete Eradication of Dysplasia		Incomplete Eradication of Dysplasia		
	107	100.0%	82	76.6%	25	23.4%	*p*-value^b^
Patient Characteristics							
Race							
White	103	96.3%	80	97.6%	23	92.0%	0.232
Other	4	3.7%	2	2.4%	2	8.0%	
Sex							
Male	93	86.9%	69	84.1%	24	96.0%	0.180
Female	14	13.1%	13	15.9%	1	4.0%	
Age	^c^64	(58-72)	^c^ 64	(57–72)	^c^ 66	(59–76)	0.336
BMI	^c^ 29.1	(25.5–32.6)	^c^ 30.5	(26.5–33.1)	^c^ 27.45	(24.5–30)	0.045
Dysplasia							
HGD	44	41.1%	30	36.6%	14	56.0%	0.084
LGD	63	58.9%	52	63.4%	11	44.0%	
Comorbidities				0.0%			
GERD	75	70.1%	60	73.2%	15	60.0%	0.208
Hyperlipidemia	26	24.3%	20	24.4%	6	24.0%	0.968
Diabetes	25	23.4%	18	22.0%	7	28.0%	0.532
Hypertension	59	55.1%	40	48.8%	19	76.0%	0.017
History of Tobacco Usage							
Yes	60	56.1%	43	52.4%	17	68.0%	0.350
No	33	30.8%	28	34.1%	5	20.0%	
Unknown	14	13.1%	11	13.4%	3	12.0%	
Endoscopic Treatments Received							
EMR	24	22.4%	18	22.0%	6	24.0%	0.732
Length							
Median, IQR	5	(2–7)	4	(2–7)	6	(2–8)	0.150
</= 5 cm	64	59.8%	52	63.4%	12	48.0%	0.038
> 5 cm	43	40.2%	30	36.6%	13	52.0%	
Number of RFA’s							
Median, IQR	3	(2–5)	3	(2–5)	4	(2–5)	0.066
≤ 3	62	57.9%	52	63.4%	10	40.0%	0.064
> 3	45	42.1%	30	36.6%	15	60.0%	

^a^Data presented as N (%) or median (IQR)

^b^*p*-value ≤0.05 is significant

^c^average; not total number of patients

**Table 3:** Predictors of Failure

Covariate	Incomplete Eradication of Intestinal Metaplasia						Incomplete Eradication of Dysplasia					
	Univariate Analysis			Multivariate Analysis			Univariate Analysis			Multivariate Analysis		
	OR	95% CI	*p*-value	OR	95% CI	*p*-value	OR	95% CI	*p*-value	OR	95% CI	*p*-value
Not White	1.34	0.18–9.89	0.77				3.48	0.46–26.07	0.23			
Female	2.06	0.60–7.04	0.25	4.157	0.94–18.40	0.060						
65 years or older	2.63	1.20–5.79	0.02	4.508	1.72–11.84	0.0022	2.16	0.857–5.45	0.10	Ref.	Ref.	0.10
Obese, BMI > 30	2.60	1.11–6.14	0.01				2.29	0.85–6.20	0.08			
HGD	1.19	0.55–2.58	0.67				2.21	0.89–5.47	0.09	2.151	0.80–5.80	0.13
GERD	1.50	0.65–3.45	0.34				Ref.	Ref.	0.21			
Hyperlipidemia	1.59	0.63–3.98	0.32				Ref.	Ref.	0.97			
Diabetes	1.17	0.47–2.92	0.73				1.38	0.50–3.83	0.53			
Hypertension	1.76	0.81–3.85	0.16				3.33	1.21–9.17	0.02	3.86	1.32–11.31	0.01
Tobacco Usage	2.50	0.71–8.85	0.23				1.45	0.36–5.85	0.36			
Length > 5 cm	4.03	1.78–9.09	< 0.01	7.064	2.62–19.06	< 0.01	2.60	1.04–6.51	0.04	3.08	1.12–8.46	0.03
> 3 RFA’s	2.16	0.96–4.89	0.06				1.15	0.45–2.93	0.78			
				c statistic:	0.766					c statistic:	0.761	
				r squared	0.285					r squared	0.2215	

There were no major complications from RFA therapy in our study population. Specifically, there was no stricturing or bleeding noted on follow-up EGD. Retrospective review from the medical records did not reveal any documented complications related to anesthesia/ sedation.

## Discussion

Many studies have analyzed the durability and recurrence of BE associated with RFA, but few studies to date have examined factors which affect the rate of eradication of CE-IM or CE-D with RFA. We analyzed multiple factors including patient characteristics such as age, comorbidities such as GERD, hypertension, or diabetes mellitus, risk factors such as tobacco use or duration of reflux, and endoscopic characteristics such as length of Barrett’s segment and number of treatments with RFA. The number of patients in our study population who drank alcohol was low, so this risk factor was not analyzed. In our study, the overall rate of CE-IM and CE-D was 57 and 76.6%, respectively. The rates of CE-IM and CE-D in our study are similar to other published studies, which demonstrate rates of CE-IM ranging from 41 to 67% [9]. Some other studies with higher rates of CE-IM and CE-D typically treated shorter lengths of BE than our current study [21, 22] which had 42.1% of patients with BE segment greater than 5 cm. We found that length of Barrett’s segment length greater than 5 cm was independently predictive of a higher rate for failure of complete eradication in patients undergoing RFA. Of those patients in our study with failure of CE-IM or CE-D, 56.6 and 60.0% of patients, respectively, had a pretreatment BE length of > 5 cm. Longer segments of BE have been associated with potentially more aggressive behavior and with a resultant higher risk of progression, which may explain the lower rates of CE in our study [11, 12]. These studies demonstrate similar findings with longer segments of BE associated with higher rates of eradication failure and recurrence. In addition, their finding that BE of length 4.8 vs. 3.8 cm had significantly higher recurrence after treatment correlates closely with our data showing that BE length > 5 cm predicts failure with RFA. Although the reasons for the association are unclear, a longer pretreatment segment may be a marker for more severe acid exposure and injury [12]. Recent studies also show a spatial preference for dysplasia being more common in proximal areas of the Barret’s segment [23]. In addition, recent literature studying cryotherapy as a modality for BE refractory to RFA has also revealed that RFA failure groups have longer Barrett’s segments. [24, 25]. We believe the length of Barrett’s segment ablated was the main reason for the large range of RFA sessions required for eradication of BE, as longer segments tended to require more frequent RFA sessions to achieve eradication. The choice of focal or circumferential ablation was standardized based on the protocol as discussed above, and we feel that the choice of ablation technique was not a contributing cause to the range of RFA sessions required for eradication. A greater BMI seems to be associated with longer segment of BE, however we did not find the BMI to be an independent predictor for failure in our study [26].

Another finding of our study was that having a greater number of RFA treatments was predictive of failure of CE-IM. Those patients who required more than 3 RFA treatments were significantly more likely to have failure of CE-IM. Of the 46 patients who failed to achieve CE-IM, 54.3% had greater than 3 RFA treatments. This is similar to findings from other studies such as Agoston et al., which also suggest increased number of treatments predicts failure of eradication [11] [27]. These results may actually be explained by a more aggressive neoplastic phenotype as opposed to a result of treatment, and could explain differences in measured rates of achieving CE-IM. Other notable statistically significant predictors of failure in our study included age greater than 64 years old for CE-IM. While the significance of this finding is unclear, it has been suggested that elderly people may have more prolonged exposure to carcinogens and are therefore more likely to accumulate somatic mutations [12]. Hypertension was also found to be a statistically significant predictor of failure of CE-D. We suspect that this is likely just a statistical finding related to small study size. Further evaluation is necessary to support these factors as predictors of eradication failure.

These findings are important because RFA is used commonly for the treatment of BE. Identifying factors which place patients at higher risk of not responding to RFA may also help identify individuals with a greater risk of progressing to neoplasia. Those patients with longer pretreatment BE are at greater risk of failure of complete eradication with RFA, and may benefit from a more invasive treatment approach such as EMR. Patient’s with persistent BE after greater than 3 treatments with RFA are at greater risk of failure of complete eradication, and this could be helpful for directing further therapy as continued RFA sessions may be less beneficial. At this point, other treatment modalities such as APC, cryotherapy, or EMR could be considered. Other contributing factors such as medication noncompliance or lack of appropriate follow-up should also be considered. It should also be noted that studies have shown that RFA is a cost-effective strategy for treatment of dysplastic Barrett’s esophagus [28].

Our study had limitations which should be considered when interpreting the data. The study was retrospective in nature, and data was collected entirely from our own single institution: a large, academic, tertiary-care hospital which is relatively new to the technique of radiofrequency ablation for eradication of Barrett’s Esophagus. Another limitation is that there was a moderate percentage that were lost to follow up, thus limiting the results. However, in general, our data is similar to that of other large, academic hospitals with high rates of complete eradication of both intestinal metaplasia and dysplasia. The retrospective nature of our study also makes misclassification possible. Also, some patients were lost to follow up for unknown reasons, which could affect the reported rates of eradication.

The lower eradication rate is another limitation of our study. This lower rate is likely related to the number of patients lost to follow-up, as well as the fact that our patient sample is entirely from a tertiary care referral center, likely dealing with the most complex cases.

## Conclusion

In conclusion, we have identified pathologic factors as well as endoscopic factors which are associated with a higher risk of failure to achieve CE-IM or CE-D with RFA treatment of BE. Knowledge of these predictors can help identify patients at higher risk for treatment failure and subsequent increased risk for neoplastic progression. This knowledge may be beneficial to prompt a more aggressive initial therapy to prevent unnecessary procedures or neoplastic progression.

## Abbreviations

BE: Barrett’s Esophagus
BMI: body mass index
CE: Complete eradication
CE-D: Complete eradication of dysplasia
CE-IM: Complete eradication of intestinal metaplasia
EAC: Esophageal adenocarcinoma
EMR: Endoscopic mucosal resection
ESD: Endoscopic submucosal dissection
HGD: High grade dysplasia
LGD: Low grade dysplasia
MAC: Monitored anesthesia care
RFA: Radiofrequency ablation
